# Metabolomic Selection in the Progression of Type 2 Diabetes Mellitus: A Genetic Algorithm Approach

**DOI:** 10.3390/diagnostics12112803

**Published:** 2022-11-15

**Authors:** Jorge Morgan-Benita, Ana G. Sánchez-Reyna, Carlos H. Espino-Salinas, Juan José Oropeza-Valdez, Huizilopoztli Luna-García, Carlos E. Galván-Tejada, Jorge I. Galván-Tejada, Hamurabi Gamboa-Rosales, Jose Antonio Enciso-Moreno, José Celaya-Padilla

**Affiliations:** 1 Unidad Académica de Ingeniería Eléctrica, Universidad Autónoma de Zacatecas, Jardín Juarez 147, Centro, Zacatecas 98000, Mexico; 2 Metabolomics and Proteomics Laboratory, Autonomous University of Zacatecas, Zacatecas 98000, Mexico

**Keywords:** genetic algorithm, machine learning, metabolites, type 2 diabetes

## Abstract

According to the World Health Organization (WHO), type 2 diabetes mellitus (T2DM) is a result of the inefficient use of insulin by the body. More than 95% of people with diabetes have T2DM, which is largely due to excess weight and physical inactivity. This study proposes an intelligent feature selection of metabolites related to different stages of diabetes, with the use of genetic algorithms (GA) and the implementation of support vector machines (SVMs), K-Nearest Neighbors (KNNs) and Nearest Centroid (NEARCENT) and with a dataset obtained from the Instituto Mexicano del Seguro Social with the protocol name of the following: “Análisis metabolómico y transcriptómico diferencial en orina y suero de pacientes pre diabéticos, diabéticos y con nefropatía diabética para identificar potenciales biomarcadores pronósticos de daño renal” (differential metabolomic and transcriptomic analyses in the urine and serum of pre-diabetic, diabetic and diabetic nephropathy patients to identify potential prognostic biomarkers of kidney damage). In order to analyze which machine learning (ML) model is the most optimal for classifying patients with some stage of T2DM, the novelty of this work is to provide a genetic algorithm approach that detects significant metabolites in each stage of progression. More than 100 metabolites were identified as significant between all stages; with the data analyzed, the average accuracies obtained in each of the five most-accurate implementations of genetic algorithms were in the range of 0.8214–0.9893 with respect to average accuracy, providing a precise tool to use in detections and backing up a diagnosis constructed entirely with metabolomics. By providing five potential biomarkers for progression, these extremely significant metabolites are as follows: “Cer(d18:1/24:1) i2”, “PC(20:3-OH/P-18:1)”, “Ganoderic acid C2”, “TG(16:0/17:1/18:1)” and “GPEtn(18:0/20:4)”.

## 1. Introduction

Diabetes is a chronic and progressive disease that occurs in the pancreas when it is no longer able to make a hormone known as insulin or when the body is unable to use it properly [1]. Adults numbering 537 million (20–79 years) currently live with diabetes in the world, and over 6.7 million deaths in 2021 are reported (approximately one death every 5 s) [2]. Type 2 diabetes mellitus (T2DM) is a progressive condition that is produced by relative insulin deficiencies caused by pancreatic β-cell (cells that synthesize and secrete insulin and amylin) dysfunction and insulin resistance [3]. The International Diabetes Federation (IDF) presented in 2021 that 541 million people in adulthood present a higher risk of developing T2DM [4]. As T2DM progresses, the comorbidities associated with hyperglycemia that induces renal damage directly or via hemodynamic modifications appear, which cause diabetic nephropathy or kidney disease (DN), and these are one of the most common problems developing in 30 to 40% of patients [5]. In recent years, metabolomics has been used as a novel approach for biomarker discovery and, in conjunction with genomics, can potentially provide a systemic understanding of the underlying causes of pathology, highlighting the importance of metabolomic approaches in the clinical sciences and helping provide guidance in clinical interventions. Metabolomics are a powerful and potentially high-throughput approach for biomarkers that can help provide molecular knowledge, identify therapeutic targets and improve the prevention of T2DM and its progression [6]. For a quicker adaptation of biomarker discoveries, portable and wearable technologies are aided by clever data mining, as well as deep learning and artificial intelligence inclusion [7]. Since individuals with decreased functional β-cell mass are at risk of developing T2DM, these individuals must be identified for prevention, but since in vivo detection remains unsuccessful, the use of metabolomics provides readouts of the states of this disease before symptoms appear. Gastrointestinal problems associated with metabolomics and diabetes [8], as well as other comorbidities, can be addressed by identifying novel plasma biomarkers for a loss of functional β-cell mass in the asymptomatic prediabetes stage as a solution to this problem, with non-targeted and targeted metabolomics. In this study performed on mice, Lingzi L. et al. [9] identified 1,5-anhydroglucitol as being associated with the loss of functional β-cell mass and uncovered metabolic similarities between the liver and plasma, providing insights into the systemic effects caused by early declines in β-cells; this deoxyhexose reflects the progressive decline of functional β-cell mass at the asymptomatic prediabetic stage. These findings stated a baseline to be applied in human cohorts so that they can be validated.

Another way to understand the metabolic function in the organs and the development and progression of T2DM is to match two-dimensional metabolic screening in tissue samples from key metabolic tissues such as serum, visceral adipose tissue, liver, pancreatic islets or skeletal muscle of individuals in different states of T2DM. In this way, carnitines are significantly higher in livers, while lysophosphatidylcholines were significantly lower in the muscle and serum of diabetes subjects. Other findings showed that lysophosphatidylcholines are significantly lower in the muscle and the serum of pre-diabetes subjects, and glycodeoxycholic acid was significantly higher in livers [10].

On the other hand, metabolites analyses are potent approaches for unraveling the relations between them and the progression or conditions of a particular disease, and a relation between conditions such as obesity and the progression of diabetes can be determined by major variations in lipid-related metabolites [11]. As there are more than 200,000 different metabolites in the human body, as shown in the human metabolome database 5.0 or HMDB 5.0 [12], data analyses by conventional methods prove to be inefficient and costly. The ML techniques provide a solution for this volume of data by detecting patterns and providing predictions. In order to stage novel results or predictions that are of high quality and usability, the data must be clearly supported by experts in the field and extracted by professional or scientific methods. The dataset also must be presented with the correct protocols for its liberation relative to the experiment or analyses that it was created for.

Peddinti G. et al. [13] implemented ML models based on entire metabolome datasets, and with a combination of glucose, mannose and α-hydroxybutyrate (known biomarkers commonly used as clinical risk factors) introduce predictive biomarkers, such as α-tocopherol, bradykinin hydroxyproline, X-12063 and X-13435, which are other metabolites that showed potential value in making precise predictions on the progression to type 2 diabetes. Moreover, Huang J. et al. [14] proposed a case of potential biomarkers in CKD prediction, and this case involved sphingomyelin C18:1 and phosphatidylcholine diacyl C38:0, which are identified specifically in hyperglycemic individuals.

Metabolites inclusion as part of the ML models brings new possibilities, making detection more robust and accurate. Another biomarker as a lone candidate on non-targeted urinary metabolomics is urine metabolome 3-hydroxy decanoyl-carnitine, which can be used for the identification of individuals with T2DM risks [15]. DN pathogenesis can be diagnosticated early with non-invasive biomarkers such as the base urea cycle, TCA cycle, glycolysis and amino acid metabolism, which includes lactic acid, hippuric acid, allantoin (in urine) and glutamine (in blood) (the latter are suggested as meta-analyses [16]). Valine (or betaine) and 3-(4-methyl-3-pentenyl)thiophene were associated with higher hazards with respect to end-stage kidney diseases [17]. The prognostic biomarkers given by metabolomics have the potential to uncover mechanisms in DN progression. Recent studies present potential target antigens in membranous nephropathy, with a signature of urinary peptides; this adds prognostic information to urinary albumin and implicates circulating inflammatory proteins as potential mediators of DN, demonstrating the importance of kidney bioenergetics as a modifiable factor in acute kidney injury [18].

ML has been widely used in the medical context using clinical data to detect patterns and/or predict different diseases, solving classification problems: using extreme learning machines on malaria parasite detection and classification [19]; using deep learning and image processing in diabetic retinopathy [20]; using Internet of Things (IoT) to provide an intelligent forensic analysis [21]; using FastAI and 1-Cycle Policy in breast cancer metastasis prediction [22]; using various multimodal models such as decision tree, logistic regression or random forest, among others, in Alzheimer’s disease progression detection [23]. The ML implementations provide information for the analyst, which can be used to perform a pre-diagnosis if the patient has a particular disease or in identifying significant features; these results as the forms of predictions or classifiers can be ratified by a medical professional, and the professional can give approval to validate or discard this pre-diagnosis.

Most of the studies presented in this work propose diverse techniques to find a relation between control subjects and prediabetes: T2DM or DN. The ones that propose an ML model use metabolomics as a complement for classification; however, there are few studies that entirely use metabolomic data as features for the classification of a disease or provide tools to predict progression. Additional studies are required to replicate and expand upon these findings in independent cohorts, such as this one.

The proposal in this study is to provide a tool for the classification and analysis of the role of metabolomics in four different stages of T2DM. The novelty of this work is to present a genetic algorithm approach that selects the most significant metabolites in each stage of T2DM progression and not an individual classification of a particular stage, as other related work proposed.

The KNN, nearest centroid and support vector machines implemented as proposed ML models inside a genetic algorithm focus on analyzing 80 patients in five different sub-datasets: Control-Prediabetes, Control-T2DM, Prediabetes-T2DM, Control-DN and T2DM-DN. The dataset for this study was acquired from the “Unidad de Investigación Médica Biomédica by the Unidad de Investigación Biomédica located in Zacatecas, México, IMSS” with information on Mexican patients. The database contains anthropometric, clinical, laboratory and metabolomic data on Mexican patients.

This work is divided into five sections. The first is this Introduction. Section 2 describes the data, models and the methodology used to carry out the development of the ensemble model and how it is validated. Section 3 shows the results obtained and a detailed analysis is included using output graphs. Finally, Section 4 shows the discussions, and in Section 5 conclusions and future work.

## 2. Materials and Methods

**Figure 1:**
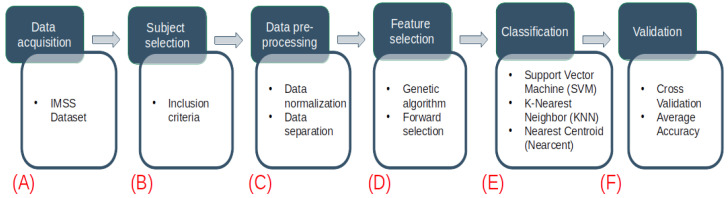
Flowchart of the proposed methodology. The blue squares refer to the data analysis methodology, while the white squares detail the task involved in each step. (**A**) The metabolomics dataset is obtained from the Unidad de Investigación Médica Biomedica. (**B**) The dataset is analyzed and new datasets are created by selecting subjects according to the criteria described in Table 1. (**C**) The data are normalized by probabilistic quotient normalization (PQN) and the dataset’s observations are analyzed and separated in three groups: Control-Prediabetes, Control-Diabetes and Control-DN. (**D**) The use of genetic algorithms is implemented to extract the main data features. (**E**) Using the main features for prediabetes, diabetes and diabetic nephropathy detection in patients, several models are generated using support vector machines, k-nearest neighbor and nearest centroid. (**F**) The validation of our results is carried out using different metrics (cross-validation by GALGO and average accuracy) to determine which of the models has the best performance.

The methodology of this study consists of six stages, as shown in Figure 1 and explained as follows: The first stage describes the dataset used (Figure 1A). In the second stage, a preliminary analysis of the data was performed by selecting subjects according to given inclusion criteria (Figure 1B). Subsequently, the data from the dataset were separated into three groups: Control-Prediabetes, Control-Diabetes and Control-DN (Figure 1C). In the fourth stage, feature selection using a genetic algorithm is implemented (Figure 1D). In the fifth stage, machine learning models (support vector machines, k-nearest neighbor and nearest centroid) were developed using the main features of the previous stage (Figure 1E). Finally, the models were validated by taking into consideration different metrics (accuracy, sensitivity, specificity and AUC) to determine the performance of our models (Figure 1F).

### 2.1. Sample

The methodology implemented in order to obtain data of each of the 80 patients and 717 metabolomic data is presented in the following subsections: Sample Preparation; Quality Control (QC) and Quality Assurance (QA); Ultra-performance Liquid Chromatography (UPLC)—Mass Spectrometry Method for Lipid Separation and Data processing.

#### 2.1.1. Sample Preparation

Thawed plasma measuring 100 μL in ice was extracted with 300 μL of precooled isopropanol (LCMS grade, Honeywell, Charlotte, NC, USA), vortexed for 1 min and incubated at −20 °C for 1 h for protein precipitation. Subsequently, the extraction mixture was centrifuged at 15,800× *g* for 15 min, and supernatants were collected. For the analysis, each aliquot was transferred into LC vials and diluted to 1:20 with a mixture of isopropanol/acetonitrile/water (2:1:1, *v*:*v*:*v*). Sample preparation and analysis orders were randomized to ensure no systematic bias was present.

#### 2.1.2. Quality Control (QC) and Quality Assurance (QA)

The instrument was subjected to maintenance in the chromatography system: the mass analyzer. The sample cone and ion sources were cleaned before the analysis. Calibration and manual tuning were also performed before running samples. A pool of human plasma from every sample in the study served as a technical replica for the entire experiment for QC. The overall variability was established by determining the relative standard deviation (RSD) for all endogenous metabolites present in 100% in the QC process. Experimental samples were randomized across the platform and run with ten QC samples at the start (column equilibration), and one QC sample was acquired for every ten samples.

#### 2.1.3. Ultra-Performance Liquid Chromatography (UPLC)—Mass Spectrometry Method for Lipid Separation

The analysis was performed using an ACQUITY UPLC I-Class (Waters Corp., Milford, MA, USA) coupled to an XEVO-G2 XS quadrupole time-of-flight (ToF) mass spectrometer (Waters, Manchester, NH, USA) with an electrospray ionization source. The samples were analyzed in the positive mode (electrospray ionization). A UPLC CSH C18 column (2.1 × 100 mm, 1.7 µm) with a binary gradient elution of solvents was utilized, as represented in Table 2.

The injection volume was five microliters. Data were acquired using positive electrospray ionization modes with the capillary voltage set to 3.2 kV, the cone voltage to 40 eV and the source temperature to 120 °C. The desolvation gas was nitrogen, with a flow rate of 900 L/h and source temperature of 550 °C. Data were acquired from a range of *m*/*z* 50–1200 in the MSE mode in which the collision energy alternated between low energy (6 eV) and high energy (ramped from 10 to 40 eV).

#### 2.1.4. Data Processing

Raw data were processed under default settings as a Unifi Export Package (.uep), which was processed in Progenesis QI (version 2.3, Waters, Milford, MA, USA). Alignment was performed using a retention time range within 0.5–14 min to avoid interference with blank peaks. A peak width of 0.06 s was used. Deconvolution was automatically performed, considering M + H, M + Na and M + NH4 as adducts; manual inspection was performed after, eliminating features with incorrect alignments in chromatograms and neutral masses. An excel file was exported and a signal-to-noise ration was calculated for each sample based on the blank features, and all features with a signal-to-noise ratio (S/N) < 3 in 80% of samples were eliminated. In addition, RSD was calculated by taking QC features as medians, and RSD values > 20% were additionally removed.

#### 2.1.5. Metabolite Identification

Putative annotations were assigned based on accurate mass and fragmentation patterns. Metabolite annotations were determined using the metascope plugin in Progenesis QI, mass measurements were taken with less than 10 ppm in error and fragmentation spectrum matching (when MSMS data were available) was conducted using the HMDB 5.0 [12], LipidMaps [24] and METLIN [25] databases. The top metabolite annotation was selected when the progenesis metascope score was >30.

### 2.2. IMSS Dataset

The dataset used in this study was provided by the Unidad de Investigación Biomedica located in Zacatecas, Mexico, which is incorporated into the IMSS. All Mexican patients signed an informed consent letter, and the data included in the IMSS dataset meet the R-2017-785-131 dictum approval according to the protocol “Análisis metabolómico y transcriptómico diferencial en orina y suero de pacientes prediabéticos, diabéticos y con nefropatía diabética para identificar potenciales biomarcadores pronósticos de daño renal” (differential metabolomic and transcriptomic analysis in the urine and serum of prediabetic, diabetic and diabetic nephropathy patients to identify potential prognostic biomarkers of renal impairment), which complies with the criteria approved by the National Committee for Scientific Research and Ethics and follows the international ethical standards of the Helsinki convention for research studies in humans. The IMSS dataset has information on metabolomic, anthropometric, clinical and laboratory tests. These assessments can be combined to measure the progression of prediabetes, diabetes and diabetic nephropathy.

### 2.3. Data Inclusion

The IMSS dataset includes 375 patients and 842 features. From this dataset, a filter was applied to select only patients and features that met the inclusion criteria indicated in Table 1. The resulting filtered dataset (FDS), after applying the inclusion criteria listed in Table 1, contains information corresponding to 80 patients (42 female/38 male), such as age (52.34 ± 10.45), metabolomics and diagnosis (20 patients positive for prediabetes, 20 patients positive for T2DM, 20 patients positive for DN and 20 control patients).

### 2.4. Data Normalization

The normalization implemented in this study is probabilistic quotient normalization (PQN) and is conducted as follows: For each function, the output mean is calculated over all samples. Then, a reference vector is generated. The median between the resulting reference vector and each sample is calculated, obtaining a vector of related coefficients. Then, each sample is divided by the mean value of the vector of coefficients; this mean value is different for each sample. The purpose of PQN is to account for concentration changes of some metabolite characteristics that affect limited regions of the data. The PQN approach assumes that changes in the concentrations of individual analytes influence only parts of the spectra, while changes in the overall concentration of a sample influence the entire spectrum. In contrast to integral normalization, which assumes that the total integral, covering all signals, is a function of dilution only, PQN instead assumes that the intensity of most signals is a function of dilution only. Therefore, a most likely quotient between the signals of the corresponding spectrum and a reference spectrum is calculated as a normalization factor, which replaces the total integral as a marker of the sample’s concentration. This most likely quotient for a specific spectrum can be derived from the distribution of signals from a spectrum divided by the corresponding signal from a reference spectrum [26]:

(1) $$I\left(i\right)=\frac{I^{old}\left(i\right)}{\sum_{k}{\left(\int_{j_{k}^{l}}^{j_{k}^{u}}{\left(I\left(x\right)\right)}^{n}dx\right)}^{\frac{1}{n}}}$$

where $I^{old}\left(i\right)$ and I(i) are the intensities of variable *i*, which is the spectral feature, wavelength, bin and chemical shift. Before and after normalization, *k* is an index of the spectral regions used for normalization, $j_{k}^{l}$ and $j_{k}^{u}$ are the lower and upper borders, respectively, of spectral region *k*, for which the power *n* of intensities I(x) is integrated [26].

### 2.5. Feature Selection

The dataset used to perform this study has 717 different metabolites, and each has a potential significance to become a biomarker or part of it to solve a classification problem; nevertheless, this task could become computationally expensive and complex to process. With genetic algorithms, this complex task can be performed and solved. GALGO [27] is a GA implemented in this study as the R package software used to perform feature selections in 5 sets of this article (Control-Prediabetes, Control-T2DM, Prediabetes-T2DM, Control-DN and T2DM-DN). For this study, GA creates an initial population of chromosomes comprising random sets of metabolites. The fitness of the chromosomes is evaluated by comparing their ability to correctly detect each stage in the progression of T2DM (control → prediabetes → T2DM → DN).

Depending on the obtained fitness score, the chromosome population continues to be replicated and the chromosomes crossover and mutate, as the fittest chromosomes will produce next-generation offspring. The process only stops when it meets the goal criteria (in this study, it is set at 1) or when the bigbangs (iterations) reach the limit proposed (3 times the number of metabolites in this case rounded to 2300 in all GALGO implementations). The GA blast (the implementation of the GALGO model) output is then submitted to a forward selection process to obtain the model that performed best (could be one or more); then, this model or set of features is ready for utilization in an ML model. Forward selection is widely used in genetic algorithms as a complement to presenting the best possible model output of GALGO implementations, such as the following example: Alzheimer’s [28], COVID-19 [29] or diabetic retinopathy [30].

GALGO allows the use of different model criteria or parameters. In this study, the k-nearest neighbors (KNN), nearest centroid (NEARCENT) and support vector machines (SVM) were configured, as shown in Table 3.

### 2.6. Model Development

Once the main features from different partitioned datasets, described in Section 2.5, have been selected, the ML models are developed. This process is used to ensure that the training and testing results are as accurate as possible, avoiding overfitting or underfitting; the models will be paired with the ones used in GALGO implementations. In this way, the selection will be consistent with the results of the ML implementations, as is presented in Table 4.

### 2.7. K-Nearest Neighbors

KNN is one of the most fundamental classification methods and is widely used when there is little to no prior knowledge about the distribution of the data. In this study, as there is no certainty about the classification prior to the implementation (only a clear separation of the stages of the T2DM progression), a KNN implementation can provide a clear path to validate this separation and to obtain a discriminant analysis of the data [31]. The Euclidean distance between a test sample and the specified training samples is commonly defined by this model. The Euclidean distance between sample $x_{i}$ and $x_{l}$ (l=1,2,⋯,n) is defined as follows:

(2) $$d\left(x_{i},x_{l}\right)=\sqrt{{\left(x_{i1}x_{l1}\right)}^{2}+{\left(x_{i2}x_{l2}\right)}^{2}+\cdots +{\left(x_{ip}x_{lp}\right)}^{2}},$$

where $x_{i}$ is an input sample with *p* features ($x_{i1},x_{i2},\cdots ,x_{ip}$), *n* is the total number of input samples (i=1,2,⋯,n) and *p* is the total number of features (j=1,2,⋯,p).

### 2.8. Nearest Centroid

The nearest centroid is one of the simplest classifiers; nevertheless, it is capable of classifying data without any feature selection (for example, raw mass spectra [32]). In addition, it is extremely fast and requires low computational power, provides a baseline for the evaluation of feature selection algorithms and allows testing a number of algorithms that were previously inapplicable. NEARCENT and KNN provide similar approaches when there is limited knowledge on the distribution. In this study, they provide validations for the classifications results relative to one another.

(3) $$\vec{\mu_{\ell }}=\frac{1}{|C_{\ell }|}\sum_{i\in C_{\ell }}x_{i}$$

Given the labeled training samples $\left(\vec{x_{1}},y_{1}\right)$, ⋯,$\left(\vec{x_{n}},y_{n}\right)$ with class labels $y_{i}\in Y$, the per-class centroids ${\vec{\mu }}_{\ell }=\frac{1}{|C_{\ell }|}\sum_{i\in C_{\ell }}{\vec{x}}_{i}$ are computed, where $C_{\ell }$ is the set of the indices of samples belonging to class ℓ∈Y.

### 2.9. Support Vector Machines

SVM is included as it is robust and precise for solving binary classification ML problems. This model uses the theory of Structural Risk Minimization to maximize its prediction accuracy and procures avoiding data overfitting [33]. This model can use a wide variety of standard or custom kernels. The radial kernel support vector machine model used in this study fits the closest observations into the new observation, grouping them (similar processes as KNN) based on how much they influence the output of the set classifier for multiple hyperplanes. This kernel has been proven to be one of the most accurate kernals for solving nonlinear separation problems [34].

The radial basis function kernel is defined as follows:

(4) $$K\left(x,x^{\prime }\right)=e^{\frac{-||x-x^{\prime }{\left|\right|}^{2}}{2\sigma^{2}}},$$

where *x* and $x^{\prime }$ are original observations and new observations, respectively [35].

### 2.10. Implementation

All models and methodology were implemented in R, which is a well-known open-source software validated by the scientific community, as well as the following packages:

- Genetic algorithms were implemented using “galgo 1.4” [27].
- For support vector machines, “caret” was used [36].

## 3. Results

The methodology proposed in Figure 1, presents the process followed in this study in six steps: data acquisition, subject selection, data pre-processing, feature selection, classification and validation. A description of the methodology to obtain the sample (Section 2.1) included the following: Sample Preparation (Section 2.1.1); Quality Control (QC) and Quality Assurance (QA) (Section 2.1.2); Ultra-performance Liquid Chromatography (UPLC)—Mass Spectrometry 158 Method for Lipidomic Analysis (Section 2.1.3); data processing (Section 2.1.4); and metabolite identification (Section 2.1.5). The data normalization process is presented in Section 2.4.

The obtained dataset is described in Section 2.2 with the inclusion criteria presented in Table 1. Data inclusion is provided in Section 2.3. After the data inclusion process, the feature selection process begins with the implementation of genetic algorithms in Section 2.5, and 15 runs were required to obtain each combination of the stages, as shown in Table 3. Each set of features obtained in the genetic algorithms integrates a model (Section 2.6), as presented in Table 4. The models are as follows: KNN (Section 2.7), NEARCENT (Section 2.8) and SVM (Section 2.9). Lastly, the implementation in R is presented in Section 2.10.

### 3.1. Galgo Results

The 15 runs of GALGO, with different datasets combining the samples and comparing them, provided an average accuracy, as presented in Table 5, and a group of features are presented in the next sections.

#### 3.1.1. GALGO Implementation with the Control-Prediabetes Dataset

**Figure 2:**
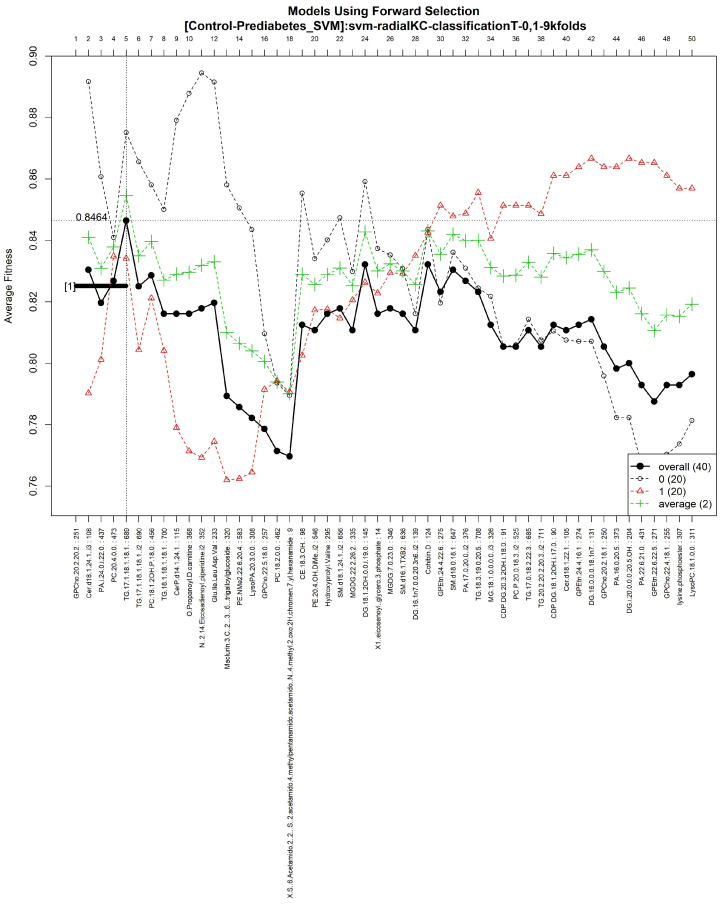
Models using forward selection methodology, with the Control-Prediabetes dataset. The solid black line represents the most compact and accurate model. The vertical axis shows the classification’s accuracy. The horizontal axis represents the ordered features, each of which corresponds to a number and a specific feature.

**Figure 3:**
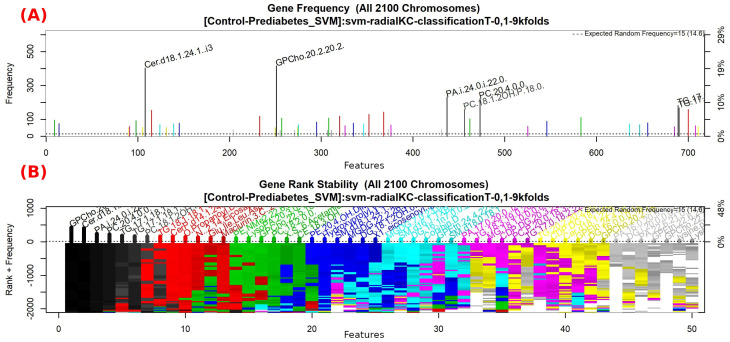
Gene frequency and gene rank stability in the models are ascertained by applying GA with SVM to select the main features from the Control-Prediabetes dataset. (**A**) The gene frequency allows observing the number of times each feature appears in the models. (**B**) The gene rank allows observing the stability and frequency of each feature within the models and orders them by rank.

**Figure 4:**
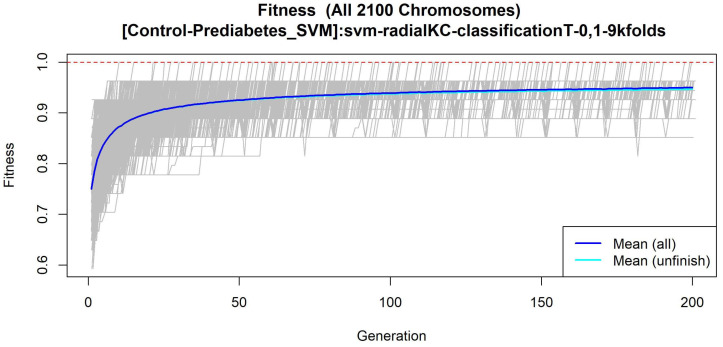
Evolution of maximum fitness scores over generations, using the Control-Prediabetes dataset. The vertical axis represents the fitness score, while the horizontal axis represents a given generation. The solid blue line represents the average fitness across all models. The average unfinished fitness plotted as a solid cyan line represents the average worst case expectation for all failed searches in a given generation. The red dotted line shows the established GA fitness goal.

The features obtained by GALGO with the SVM classification model and the forward selection best model, in this case model 1, are presented in Table 6 and in Figure 2, with an average accuracy of 0.8464, as shown in Table 5. Derived from low-quantity data against the large quantity of features included in the model, these results prove that the resultant metabolites included are extremely significant, as is presented in the gene rank’s stability (Figure 3) and fitness (Figure 4).

#### 3.1.2. GALGO Implementation with the Control-T2DM Dataset

**Figure 5:**
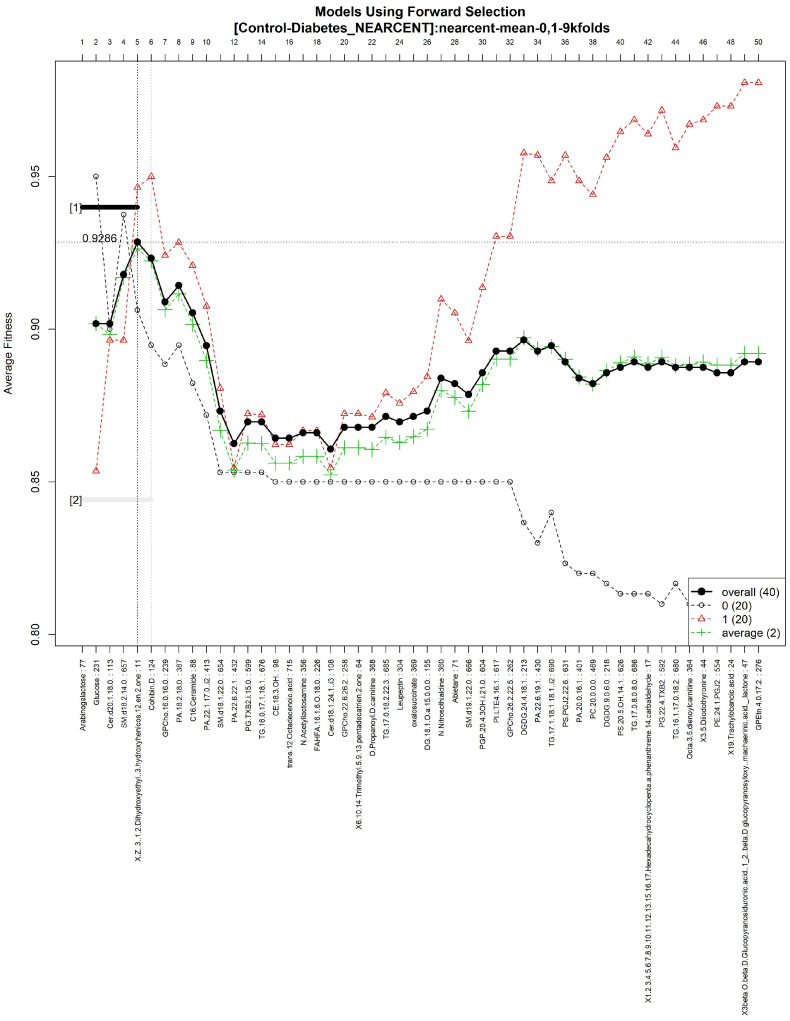
Models using the forward selection methodology, with the Control-T2DM dataset. The solid black line represents the most compact and accurate models. The vertical axis shows the classification accuracy. The horizontal axis represents the ordered features, each of which corresponds to a number.

**Figure 6:**
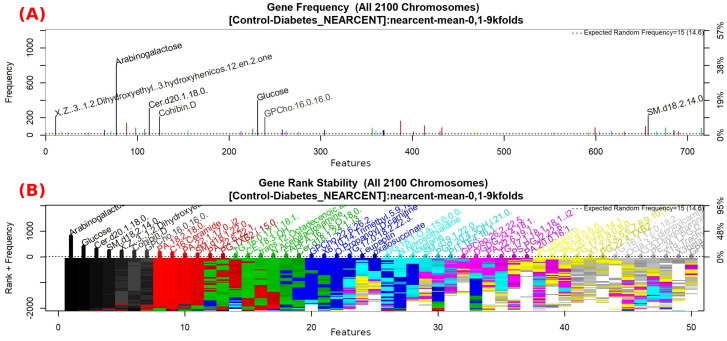
Gene frequency and gene rank stability in the models are ascertained by applying GA with NEARCENT to select the main features from the Control-T2DM dataset. (**A**) Gene frequency allows observing the number of times each feature appears in the models. (**B**) Gene rank allows observing the stability and frequency of each feature within the models and orders them by rank.

**Figure 7:**
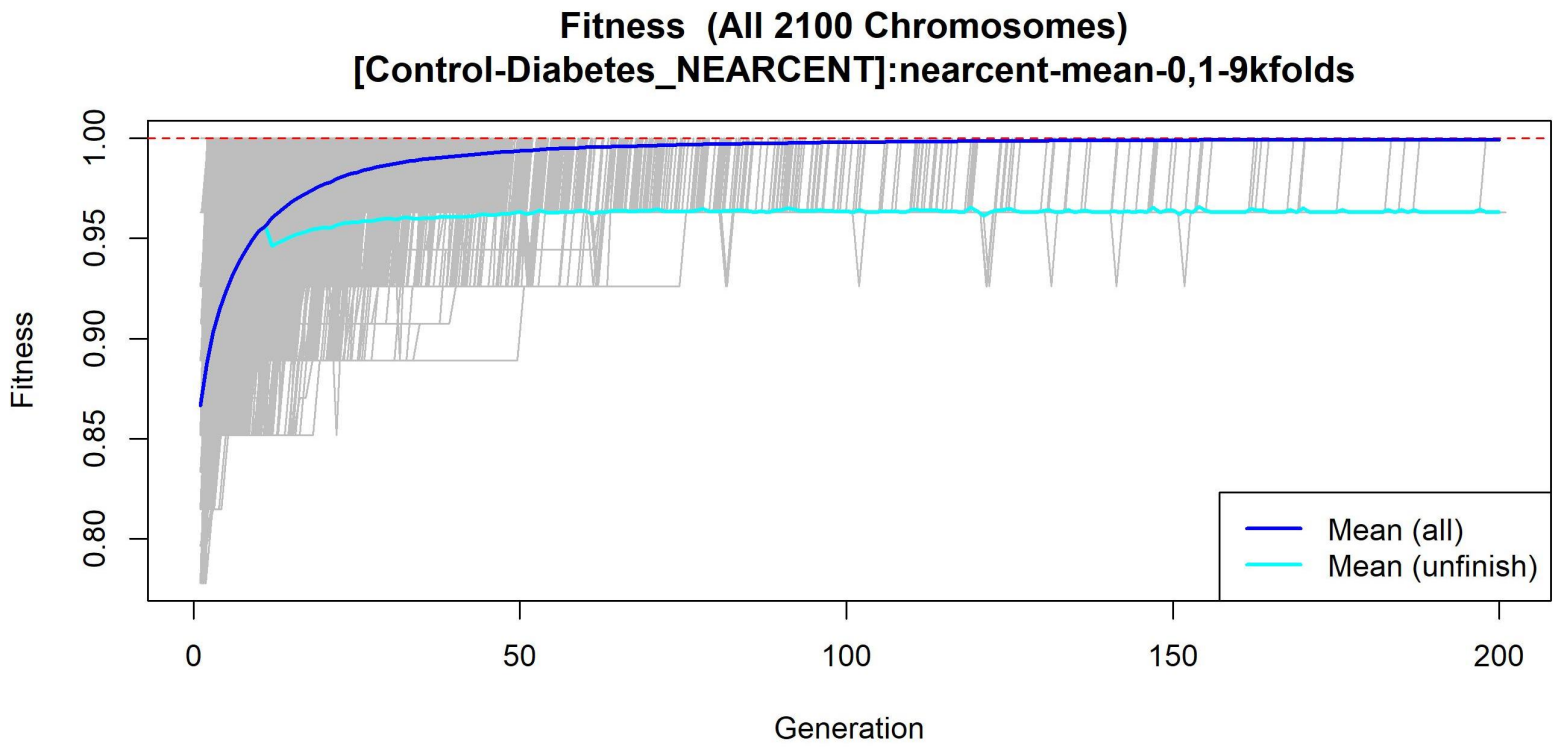
Evolution of maximum fitness scores over generations, using the Control-T2DM dataset. The vertical axis represents the fitness score, while the horizontal axis represents a given generation. The solid blue line represents the average fitness across all models. The average unfinished fitness is plotted as a solid cyan line and represents the average worst case expectation for all failed searches in a given generation. The red dotted line shows the established GA goal fitness.

The features obtained by GALGO with the NEARCENT classification method and the forward selection best model, in this case model 1, are presented in Table 7 and in Figure 5, with an average accuracy of 0.9286 (as shown in Table 5). Derived from low-quantity data against the large quantity of features included in the model, these results prove that the resultant metabolites included are extremely significant, as presented in the gene rank’s stability (Figure 6) and in fitness (Figure 7).

#### 3.1.3. GALGO Implementation with the Prediabetes-T2DM Dataset

**Figure 8:**
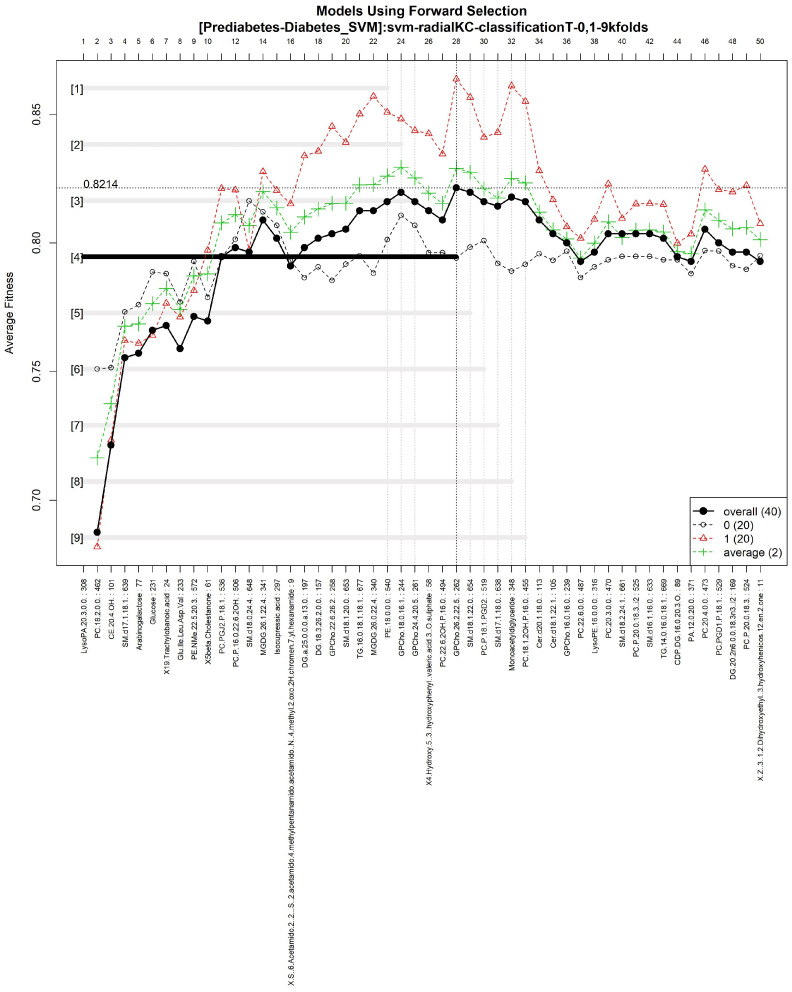
Models using forward selection methodology, with the Prediabetes-T2DM dataset. The solid black line represents the most compact and accurate model. The vertical axis shows the classification accuracy. The horizontal axis represents ordered features, each of which corresponds to a number.

**Figure 9:**
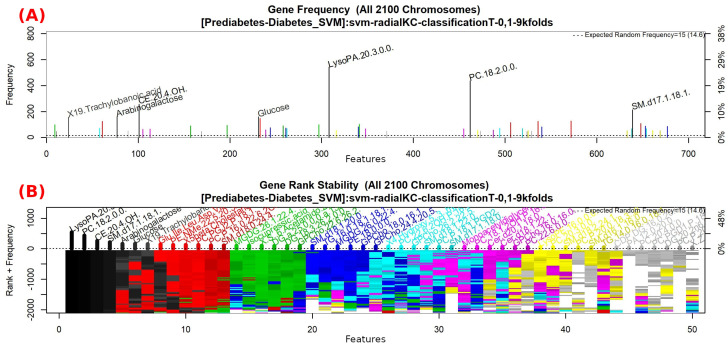
Gene frequency and gene rank stability in the models are ascertained by applying GA with SVM to select the main features from the Prediabetes-T2DM dataset. (**A**) Gene frequency allows observing the number of times each feature appears in the models. (**B**) Gene rank allows observing the stability and frequency of each feature within the models and orders them by rank.

**Figure 10:**
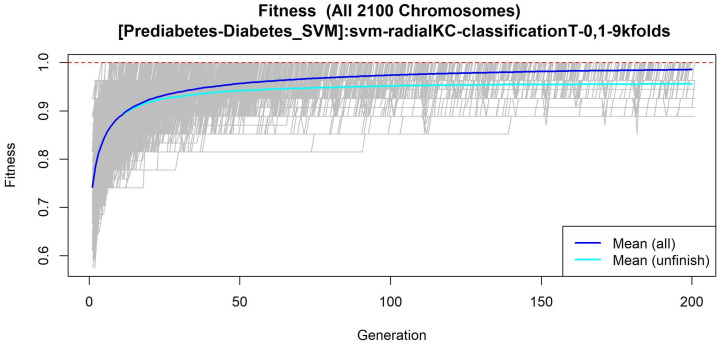
Evolution of maximum fitness scores over generations, using the Prediabetes-T2DM dataset. The vertical axis represents the fitness score, while the horizontal axis represents a given generation. The solid blue line represents the average fitness across all models. The average unfinished fitness plotted as a solid cyan line represents the average worst case expectation for all failed searches in a given generation. The red dotted line shows the established GA goal fitness.

The features obtained by GALGO with the SVM classification method and the forward selection best model, in this case model 4, are presented in Table 8 and in Figure 8, with an average accuracy of 0.8214, as shown in Table 5. Derived from low-quantity data against the large quantity of features included in the model, these results prove that the resultant metabolites included are extremely significant, as presented in the gene rank’s stability (Figure 9) and fitness (Figure 10).

#### 3.1.4. GALGO Implementation with the Control-DN Dataset

**Figure 11:**
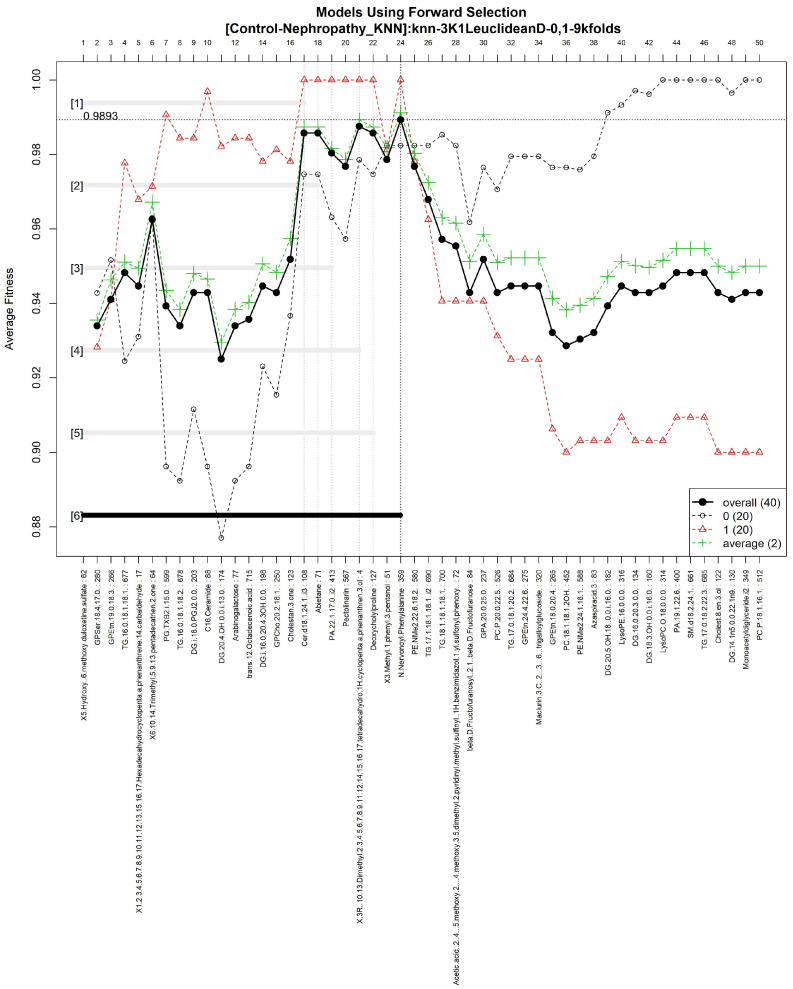
Models using forward selection methodology, with the Control-DN dataset. The solid black line represents the most compact and accurate models. The vertical axis shows the classification accuracy. The horizontal axis represents the ordered features, each of which corresponds to a number.

**Figure 12:**
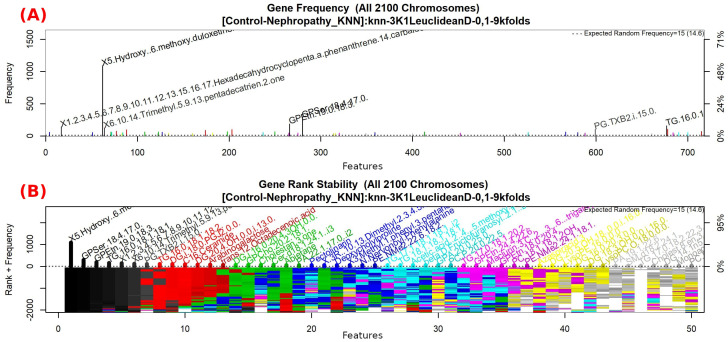
Gene frequency and gene rank stability in models are ascertained by applying GA with KNN to select the main features from the Control-DN dataset. (**A**) Gene frequency allows observing the number of times each feature appears in the models. (**B**) Gene rank allows observing the stability and frequency of each feature within the models and orders them by rank.

**Figure 13:**
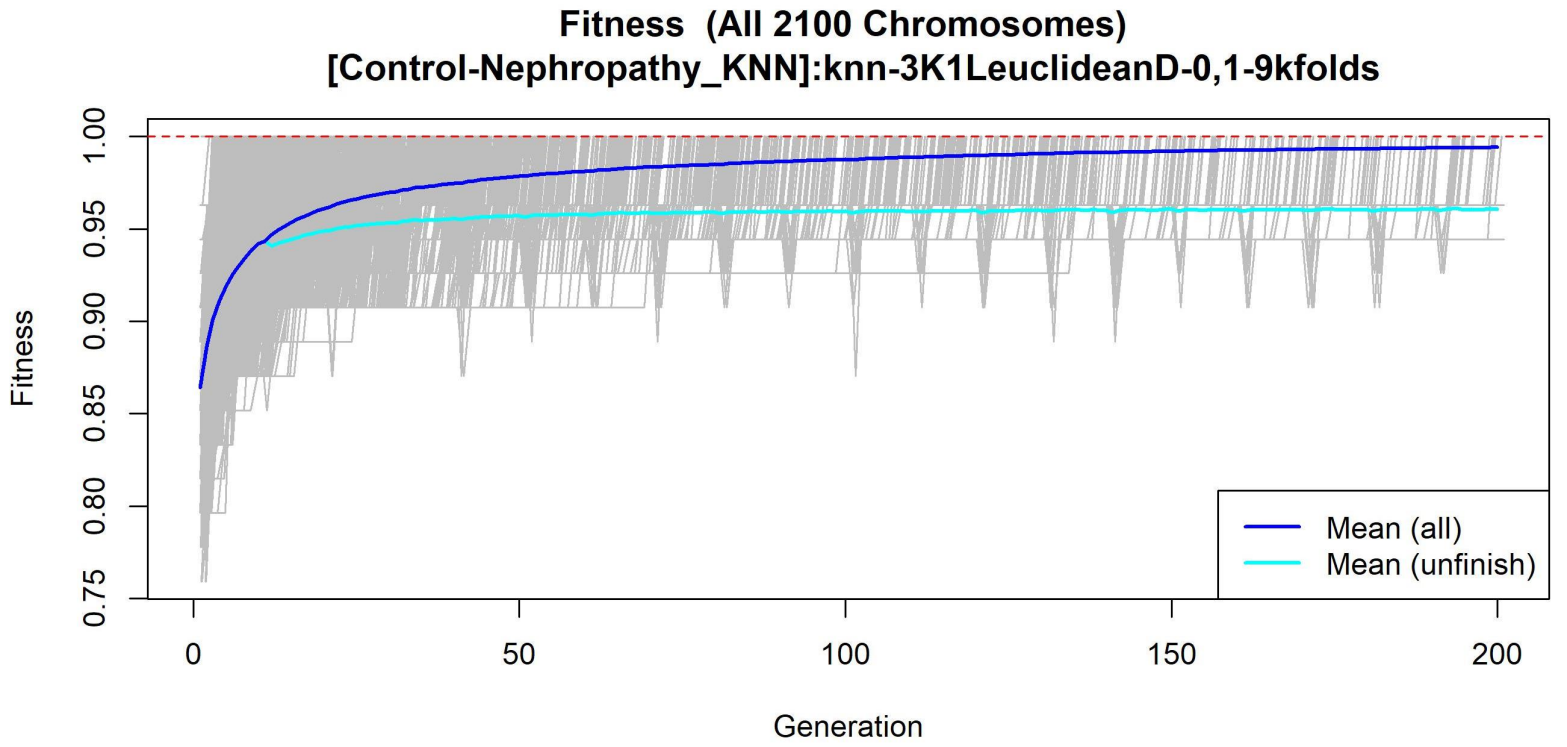
Evolution of maximum fitness scores over generations, using the Control-DN dataset. The vertical axis represents the fitness score, while the horizontal axis represents a given generation. The solid blue line represents the average fitness across all models. The average unfinished fitness plotted as a solid cyan line represents the average worst case expectation for all failed searches in a given generation. The red dotted line shows the established GA goal fitness.

The features obtained by GALGO with the KNN classification method and the forward selection best model, in this case model 6, are presented in Table 9 and in Figure 11 with an average accuracy of 0.9893, as shown in Table 5. Derived from low-quantity data against the large quantity of features included in the model, these results prove that the resultant metabolites included are extremely significant, as presented in the gene rank’s stability (Figure 12) and fitness (Figure 13).

#### 3.1.5. GALGO Implementation with the T2DM-DN Dataset

**Figure 14:**
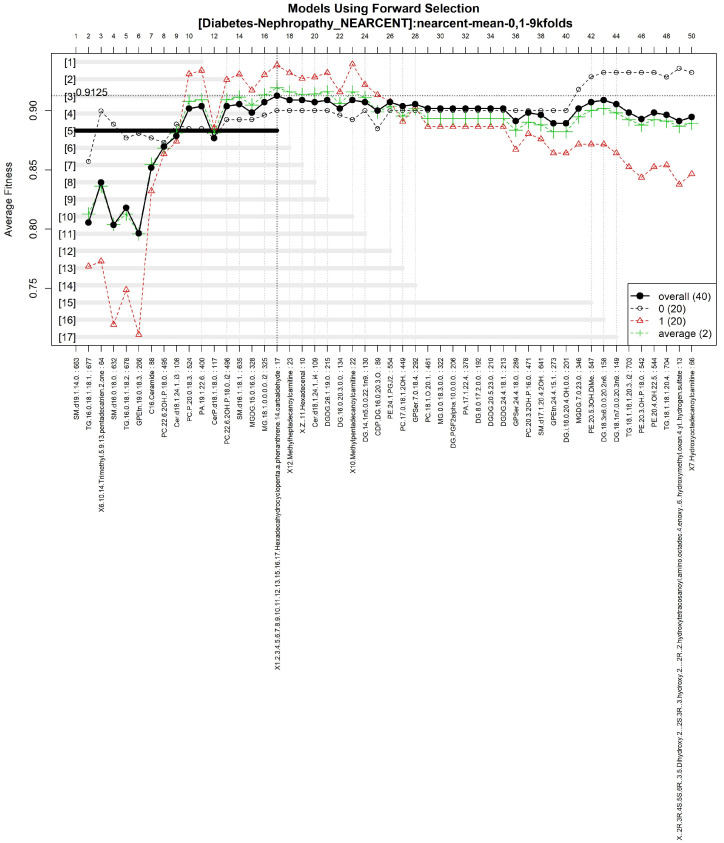
Models using forward selection methodology, with the T2DM-DN dataset. The solid black line represents the most compact and accurate model. The vertical axis shows the classification accuracy. The horizontal axis represents the ordered features, each of which corresponds to a number.

**Figure 15:**
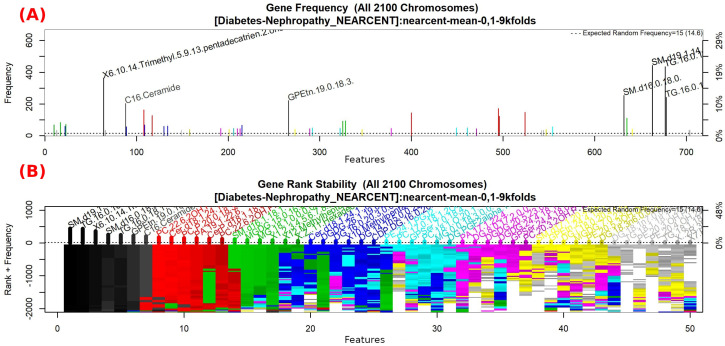
Gene frequency and gene rank stability in the models are ascertained by applying GA with NEARCENT to select the main features from the T2DM-DN dataset. (**A**) Gene frequency allows observing the number of times each feature appears in the models. (**B**) Gene rank allows observing the stability and frequency of each feature within the models and orders them by rank.

**Figure 16:**
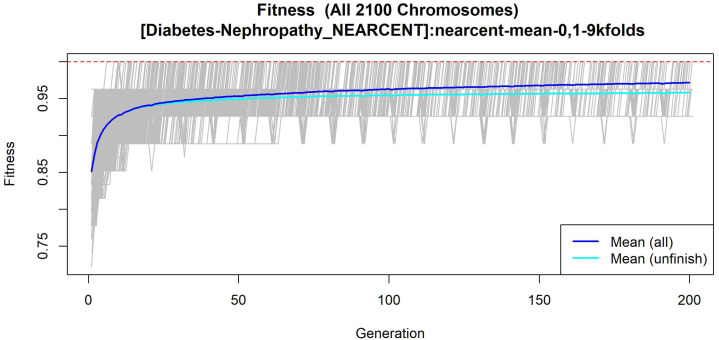
Evolution of maximum fitness scores over generations, using the T2DM-DN dataset. The vertical axis represents the fitness score, while the horizontal axis represents a given generation. The solid blue line represents the average fitness across all models. The average unfinished fitness is plotted as a solid cyan line and represents the average worst case expectation for all failed searches in a given generation. The red dotted line shows the established GA goal fitness.

The features obtained by GALGO with the NEARCENT classification method and the forward selection best model, in this case model 5, are presented in Table 10 and in Figure 14, with an average accuracy of 0.9125, as shown in Table 5. Derived from low-quantity data against the large quantity of features included in the model, these results prove that the resultant metabolites included are extremely significant, as presented in the gene rank’s stability (Figure 15 and fitness (Figure 16).

Comparing Table 11, Table 12, Table 13, Table 14 and Table 15, the metabolite addressed as “Cer(d18:1/24:1) i2” was found in three out of five stages of T2DM, Control-Prediabetes, Control-ND and T2DM-ND. The metabolite “PC(20:3-OH/P-18:1)” was found in Control-Prediabetes and Prediabetes-T2DM. The metabolite “Ganoderic acid C2” was found in Control-T2DM and Prediabetes-T2DM. The metabolites “TG(16:0/17:1/18:1)” and “GPEtn(18:0/20:4)” were found in Control-ND and T2DM-ND. This findings were obtained by matching the results of the most significant metabolites presented in each table of common metabolites found.

## 4. Discussion

The use of ML is proven to be an effective classifier and feature selection tool; however, the metabolomic community has some concerns with the lack of explanation on where does this biomarker’s significance come from. There are some methods that unveil these doubts; the statistical validation, for example, has the most widely known validator, which is the AUC or area under the curve [37]. In this proposal, this metric however will not be used; instead, the strict use of average accuracies in the feature selection with genetic algorithms is proposed as it comes from a validator given by the forward selection method provided by GALGO.

The proposed selection and model implementation shows the potential to establish significant metabolites in each stage of the disease described. The progression asseveration can be made as features collide and the model’s products of the features and the ML method can be used for fitting classifiers. As the metabolomics obtained are mainly set from serum samples, the families of lipids involved in the features obtained reveal metabolites of potential risk and more specifically propose a base to create a support tool for personalized diagnosis.

To establish a connection in each stage, the five different sets were created within a previous classification and direct transition, so it can be asseverated that there will be a statistical relation between each stage. The proposal provides a selection of features independently of the type of data selected, as in this case of metabolomics, which has been a wide field to cover but is a powerful ally for diagnosing a disease or for identifying progression.

The identification of metabolites as a potential or candidate biomarker of the incidence of DN in hyperglycemic subjects can be performed with intelligent feature selection [14]; nevertheless, in the case of LASSO, the biomarkers obtained can be diverse depending not only on the type of metabolomics in the dataset; since genetic algorithms prove the superiority and diversity of models against LASSO, new approximations can be made. The prediction of the progression from T2DM to DN remains difficult, even with potential biomarkers, with respect to detecting one or another, and novel biomarkers are needed to for detecting the progression of the disease. Nevertheless, ML methods can detect potential biomarkers that could otherwise escape identification using a conventional statistical method. Even identifying a potential biomarker such as the urinary 1-methylpyridin-1-ium (NMP) [38] with non-targeted metabolites still needs other features as complements in order to be marked as clinically usable; in contrast, a group of significant metabolites can be useful for the detection of each stage of the disease. Limiting the number of features to find strong predictors could highlight differences in pathways leading to accurate predictions on more specific populations and provide novel clues to lead to features of strong significance [39]; however, there is a need to have sufficient observations to make a model that does not overfit, and even with this, it becomes difficult in T2DM’s progression detection as there is no certainty as to when this disease will show symptoms or metrics that can be registered, as it can takes decades to develop. In cases of a small number of observations and even with a leave-one-out cross validation that seems to overfit, feature selection can stays stable with good average accuracy values (over 0.82 in this study) when there are plenty of features (717 in this case).

The metabolite’s biomarker identification can widely assist in understanding progression and in obtaining a more precise assumption on how a disease works in a specific part of the human body or how it can progress in terms of increasing risks towards developing a comorbidity. For example, in gestational diabetes, a series of metabolites can identify potential risks in developing T2DM by only perceiving the variations and finding patterns with ML and data-mining techniques [40].

In comparison with clinical or anthropometric data, metabolite data are presented as complements of models; however, the metabolites presented in this study can perform better than clinical and anthropometric data [41]. This behavior can be explained by the use of genetic algorithms as selectors and the proper preselection and prediction of the most important metabolites or family-related metabolites with respect to the disease.

The methods that best performed in the GALGO implementations were as follows: In the Control-Prediabetes dataset, SVM had 0.8464 with respect to the average accuracy and five features (to see features, view Table 6); in the Control-T2DM dataset, NEARCENT had 0.9286 with respect to the average accuracy and five features (to see features, view Table 7); in the Prediabetes-T2DM dataset, SVM had 0.8214 with respect to the average accuracy and 28 features (to see features, view Table 8); in the Control-DN dataset, KNN had 0.9893 with respect to the average accuracy and 24 features (to see features, view Table 9); and in T2DM-DN dataset, NEARCENT had 0.9125 with respect to the average accuracy and 17 features (to see features, view Table 10). The comparisons were made with an average accuracy of each GALGO selection in each clasificator method implemented in each dataset (see Table 16). Since the methods that resulted in the best average accuracies were relatively small (the larger set of metabolites selected was 28 of 717), a comparable model can be established with the same quantities and measured as percentage of features selected from the total, with other related works that had less than 10 features as the final outputs [14,38,39].

## 5. Conclusions

The metabolite selection method presented in this study has the potential to classify each stage of the T2DM with superior average accuracies. These metabolites can provide potential biomarkers for classification. The comparison between each classification method provided two assertions: Even when the methods used different approaches, they have similar results in a few features, and the features repeated in two of three OR three of three methods for each dataset are those that are extremely significant. In this case, those particular metabolites presented in each dataset are significant and provide the tools for classification between stages. These particular metabolites colliding between stages can be considered potential biomarkers for progression, and these metabolites are as follows: “Cer(d18:1/24:1) i2”, “PC(20:3-OH/P-18:1)”, “Ganoderic acid C2”, “TG(16:0/17:1/18:1)” and “GPEtn(18:0/20:4)”. Further investigations are required to establish more relations or to corroborate with the observation that these metabolites are extremely significant.

As a disadvantage, the small number of observations presented limitations in this study and could not be used as part of an ML model for more validation, as this could present potential overfitting during performances (100% in AUC). More observations with the same metabolites can validate or complement this proposal. 

## Figures and Tables

**Table 1:** Inclusion criteria.

Inclusion Criteria
1. The age of the patients must be over 18 years
2. There will be no distinction in gender, education, ethnicity, race and marital status.
3. The datasets should contain only the metabolomics of each subject.
4. The dataset should distinguish controls from prediabetes, T2DM and DN.
5. The data of each feature in each subject must be complete.

**Table 2:** Chromatography conditions.

Time (min)	%A	%B	Curve
Initial	60	40	Initial
2	57	43	6
2.1	50	50	1
12	46	54	6
12.1	30	70	1
18	1	99	6
18.1	60	40	6
20	60	40	1
Column temperature 55 °C			

**Table 3:** GALGO parameters.

Model	Parameter	Value
KNN	classification.method chromosomeSize maxSolutions maxGenerations goalFitness	knn 5 2100 200 1
Nearest Centroid	classification.method chromosomeSize maxSolutions maxGenerations goalFitness	nearcent 5 2100 200 1
SVM	classification.method svm.kernel chromosomeSize maxSolutions maxGenerations goalFitness	svm radial 5 2100 200 1

**Table 4:** GALGO models—ML models.

Sub-Dataset	GALGO Model	ML Model
	knn	K-Nearest Neighbours
Control-Prediabetes	nearcent	Nearest Centroid
	svm	Support Vector Machines
	knn	K-Nearest Neighbours
Control-T2DM	nearcent	Nearest Centroid
	svm	Support Vector Machines
	knn	K-Nearest Neighbours
Prediabetes-T2DM	nearcent	Nearest Centroid
	svm	Support Vector Machines
	knn	K-Nearest Neighbours
Control-DN	nearcent	Nearest Centroid
	svm	Support Vector Machines
	knn	K-Nearest Neighbours
T2DM-DN	nearcent	Nearest Centroid
	svm	Support Vector Machines

**Table 5:** GALGO models—Average Accuracy.

Sub-Dataset	GALGO Model	ML Model	Average Accuracy
	knn	K-Nearest Neighbours	0.8143
Control-Prediabetes	nearcent	Nearest Centroid	0.8321
	**svm**	**Support Vector Machines**	**0.8464**
	knn	K-Nearest Neighbours	0.9268
Control-T2DM	**nearcent**	**Nearest Centroid**	**0.9286**
	svm	Support Vector Machines	0.9036
	knn	K-Nearest Neighbours	0.7821
Prediabetes-T2DM	nearcent	Nearest Centroid	0.7982
	**svm**	**Support Vector Machines**	**0.8214**
	**knn**	**K-Nearest Neighbours**	**0.9893**
Control-DN	nearcent	Nearest Centroid	0.9714
	svm	Support Vector Machines	0.9857
	knn	K-Nearest Neighbours	0.9054
T2DM-DN	**nearcent**	**Nearest Centroid**	**0.9125**
	svm	Support Vector Machines	0.8804

Bold text represent the model with the highest Average accuracy in each sub-dataset.

**Table 6:** Features obtained by the GALGO SVM method in the Control-Prediabetes dataset.

Result Features
“GPCho(20:2/18:1)”, “Cer(d18:1/24:1) i2”, “PA(i-19:0/20:3-2OH)”, “PC(20:3-OH/P-18:1)”, “TG(17:1/17:2/22:5)”

All features in this table were obtained with GALGO with an SVM method implementation, with 2100 bigbangs and 200 generations.

**Table 7:** Features obtained by GALGO NEARCENT method in the Control-T2DM dataset.

Result Features
“Androst-16-ene”, “Ganoderic acid C2”, “Cer(d18:2/20:4-3OH)”, “SM(d18:1/24:1) i2”, “(Z)-11-Hexadecenal”

All features in this table were obtained with GALGO with a NEARCENT method implementation with 2100 bigbangs and 200 generations.

**Table 8:** Features obtained by the GALGO SVM method in the Prediabetes-T2DM dataset.

Result Features
“lysine phosphoester”, “PC(18:1-O/20:1)”, “CE(20:4-2OH)”, “SM(d17:1/18:0)”, “Androst-16-ene”, “Ganoderic acid C2”, “12-Methylheptadecanoylcarnitine”, “Glufosinate”, “PE-NMe(22:0/18:1)”, “5alpha-Androsta-16-ene-3-ol”, “PC(PGJ2/DiMe)”, “PC(P-16:0/20:4-3OH)”, “SM(d18:0/18:1)”, “MGDG(26:0/22:4)”, “Isobehenic acid”, “(Melle-4)cyclosporin”, “DG(a-17:0/0:0/8:0) i2”, “DG(18:3/18:1/0:0)”, “GPCho(22:5/18:0)”, “SM(d18:1/18:1)”, “TG(16:0/17:1/18:1)”, “MGDG(24:1/18:1)”, “PE(15:0/18:4)”, “GPCho(16:1/16:1)”, “GPCho(24:1/22:6)”, “4-Hydroperoxycyclophosphamide”, “PC(22:6-2OH/24:0) i2”, “GPCho(24:4/20:5)”

All features in this table were obtained with GALGO with an SVM method implementation with 2100 bigbangs and 200 generations.

**Table 9:** Features obtained by the GALGO KNN method in the Control-DN dataset.

Result Features
“5beta-Cholestanone”, “GPSer(18:1/11:0)”, “GPEtn(18:0/20:4)”, “TG(16:0/17:1/18:1)”, “1,1-Dimethylbiguanide”, “5-Pentahydroxy-5-cucurbiten-11-one 3-[glucosyl-(1->6)-glucoside]”, “PG(PGF1alpha/i-16:0)”, “TG(16:0/18:1/18:1)”, “DG(i-18:0/22:6-OH/0:0)”, “Butyl methacrylate”, “DG(20:4/16:0/0:0)”, “Androst-16-ene”, “Threonyltyrosine”, “DG(a-25:0/0:0/a-13:0)”, “GPCho(20:2/18:0)”, “Cholest-8-en-3-ol”, “Cer(d18:1/24:1) i2”, “9-Decenoylcarnitine”, “PA(22:1/17:0)”, “PE(TXB2/DiMe)”, “(2Z,4E,6Z)-Decatrienoylcarnitine”, “delta-24-Cholesterol”, “3-methoxy-4-hydroxy-5-all-trans-hexaprenylbenzoate”, “N-Methylethanolaminium phosphate”

All features in this table were obtained with GALGO with a KNN method implementation with 2100 bigbangs and 200 generations.

**Table 10:** Features obtained by GALGO NEARCENT method in the T2DM-DN dataset.

Result Features
“SM(d19:0/20:3-OH)”, “TG(16:0/17:1/18:1)”, “5-Pentahydroxy-5-cucurbiten-11-one 3-[glucosyl-(1->6)-glucoside]”, “PS(PGJ2/22:6)”, “TG(16:0/18:1/18:1)”, “GPEtn(18:0/20:4)”, “Butyl methacrylate”, “PC(22:6-2OH/P-16:0)”, “Cer(d18:1/24:1) i2”, “PC(P-20:0/14:1)”, “PA(19:1/16:0)”, “CerP(d15:0/2:0)”, “PC(22:6-2OH/P-18:0)”, “SM(d16:1/18:0)”, “MG(20:4/0:0/0:0)”, “MG(18:1/0:0/0:0)”, “1,1-Dimethylbiguanide”

All features in this table were obtained with GALGO with a NEARCENT method implementation with 2100 bigbangs and 200 generations.

**Table 11:** Common metabolites found in each model for Control-Prediabetes.

KNN and NEARCENT and SVM	KNN and NEARCENT	KNN and SVM	NEARCENT and SVM
“PC(20:3-OH/P-18:1)”	“TG(17:1/18:1/18:1)”	“GPCho(20:2/18:1)”	
“TG(17:1/17:2/22:5)”	“Glufosinate”	“Cer(d18:1/24:1) i2”	
	“PC(18:1-O/20:1)”		
	“DGDG(20:5/14:0)”		
	“TG(18:0/18:1/20:2)”		
	“lysine phosphoester”		
	“TG(17:0/18:1/20:2)”		

**Table 12:** Common metabolites found in each model for Control-Diabetes.

KNN and NEARCENT and SVM	KNN and NEARCENT	KNN and SVM	NEARCENT and SVM
“Androst-16-ene”		“Cholestan-3-one”	“(Z)-11-Hexadecenal”
“Ganoderic acid C2”		“PA(18:1/18:2) i2”	
“Cer(d18:2/20:4-3OH)”		“GPA(26:2/6:0)”	
“SM(d18:1/24:1) i2”		“CE(18:2+=O)”	

**Table 13:** Common metabolites found in each model for Prediabetes-Diabetes.

KNN and NEARCENT and SVM	KNN and NEARCENT	KNN and SVM	NEARCENT and SVM
	“PC(20:3-OH/P-18:1)”	“Ganoderic acid C2”	

**Table 14:** Common metabolites found in each model for Control-Nephropathy.

KNN and NEARCENT and SVM	KNN and NEARCENT	KNN and SVM	NEARCENT and SVM
“5beta-Cholestanone”		“Cer(d18:1/24:1) i2”	“GPSer(18:1/11:0)”
“DG(a-25:0/0:0/a-13:0)”			“PC(18:0/18:1-2OH)”
“PG(PGF1alpha/i-16:0)”			
“DG(20:4/16:0/0:0)”			
“N-Methylethanolaminium phosphate”			
“GPEtn(18:0/20:4)”			
“TG(16:0/17:1/18:1)”			

**Table 15:** Common metabolites found in each model for Diabetes-Nephropathy.

KNN and NEARCENT and SVM	KNN and NEARCENT	KNN and SVM	NEARCENT and SVM
“Butyl.methacrylate”	“SM(d19:0/20:3-OH)”		
“TG(16:0/17:1/18:1)”	“GPEtn(18:0/20:4)”		
“Cer(d18:1/24:1) i2”	“PC(22:6-2OH/P-16:0)”		
“TG(16:0/18:1/18:1)”	“CerP(d15:0/2:0)”		
“1,1-Dimethylbiguanide”	“PC(22:6-2OH/P-18:0)”		
“PS(PGJ2/22:6)”	“SM(d16:1/18:0)”		
“5-Pentahydroxy-5-cucurbiten-11-one 3-[glucosyl-(1->6)-glucoside]”	“PA(19:1/16:0)”		

**Table 16:** Comparison of feature selection with related work.

Title	Feature Selection Technique	Validation Metric	Result
Machine Learning Approaches Reveal Metabolic Signatures of Incident Chronic Kidney Disease in Individuals With Prediabetes and Type 2 Diabetes [14]	LASSO	AUC	0.857
Potential progression biomarkers of diabetic kidney disease determined using comprehensive machine learning analysis of non-targeted metabolomics [38]	NON	AUC	0.775
Predictive Modeling of Type 1 Diabetes Stages Using Disparate Data Sources [39]	Repeated Optimization for Feature Interpretation	AUC	0.91
Machine Learning–Derived Prenatal Predictive Risk Model to Guide Intervention and Prevent the Progression of Gestational Diabetes Mellitus to Type 2 Diabetes: Prediction Model Development Study [40]	CatBoost tree ensembles	AUC	0.86
Data-Driven Machine-Learning Methods for Diabetes Risk Prediction [41]	Pearson Correlation, Gain Ratio, Naive Bayes and Random Forest	AUC	0.942
Interpretable machine learning-derived nomogram model for early detection of diabetic retinopathy in type 2 diabetes mellitus: a widely targeted metabolomics study [42]	Classification and Regression Tree	AUC	0.95
Environmental chemical exposure dynamics and machine learning-based prediction of diabetes mellitus [43]	Lasso	AUC	0.78
**This Work in Control-Prediabetes**	**Genetic Algorithm with GALGO-svm**	**Accuracy**	**0.8464**
**This Work in Control-T2DM**	**Genetic Algorithm with GALGO-Nearcent**	**Accuracy**	**0.9286**
**This Work in Prediabetes-T2DM**	**Genetic Algorithm with GALGO-svm**	**Accuracy**	**0.8214**
**This Work in Control-DN**	**Genetic Algorithm with GALGO-knn**	**Accuracy**	**0.9893**
**This Work in T2DM-DN**	**Genetic Algorithm with GALGO-nearcent**	**Accuracy**	**0.9125**

Bold text represent this work models with the highest Average accuracy in each sub-dataset.

## Data Availability

Not applicable.
